# Increased risk of dementia among people with a history of fractures: a systematic review and meta-analysis of population-based studies

**DOI:** 10.3389/fneur.2023.1185721

**Published:** 2023-07-20

**Authors:** Li Su, Youyou Liao, Xueqiao Liu, Xin Xie, Yujie Li

**Affiliations:** Department of Neurology, The General Hospital of Western Theater Command PLA, Chengdu, China

**Keywords:** fracture, dementia, Alzheimer's disease, epidemiology, risk factors, meta-analysis, systematic review

## Abstract

**Background:**

Emerging evidence suggests that there may be an association between a history of fractures and dementia risk, but the epidemiological findings are inconsistent. We, therefore, conducted a meta-analysis to systematically assess the risk of dementia among people with a history of fractures.

**Methods:**

We comprehensively searched four electronic databases (PubMed, Web of Science, Embase, and Cochrane Library) for relevant literature published from inception to 10 January 2023. Longitudinal observational studies that investigated the association between any type of fracture occurrence and the subsequent risk of dementia were included for qualitative and quantitative analysis. Risk estimates were pooled using fixed-effects or random-effects models according to the level of heterogeneity. The Newcastle-Ottawa scale was used to evaluate the risk of bias in the included studies.

**Results:**

A total of seven population-based studies involving 3,658,108 participants (136,179 with a history of fractures) were eventually included. Pooled results showed a significant association between fracture and subsequent risk of dementia [hazard ratio (HR) = 1.28, 95% confidence interval (CI): 1.11–1.48] in cohort studies. Patients with fractures at different sites showed a similar trend toward increased risk of subsequent dementia. No gender, age, region, duration of follow-up, study quality, or study design specificity were observed. Sensitivity analysis indicates that the current results are robust. No publication bias existed. The results were similar in the cohort study with the standardized incidence ratio (SIR) as the statistical measure (SIR = 1.58, 95% CI: 1.25–2.00) and in the case-control study (OR = 1.38, 95% CI: 1.18–1.61). Of note, the causal relationship between fracture and dementia was not demonstrated in this meta-analysis.

**Conclusion:**

People with a history of fractures are at increased risk of developing dementia. Enhanced screening and preventive management of dementia in people with a history of fractures may be beneficial.

## Introduction

Dementia is a syndrome characterized by progressive impairment in cognitive function, memory loss, and a decline in the ability to perform daily activities (1). Approximately 55 million people worldwide currently have dementia, with up to 10 million new cases each year, and the number of people living with dementia is projected to reach 139 million by 2050 (2). Dementia is one of the greatest public health challenges in modern society and places a huge burden on society, families, and individuals; the total global societal cost of dementia is estimated to be as high as US$1.3 trillion in 2019 (2–4). Unfortunately, there is no established effective disease-modifying treatment for dementia, so prevention of dementia is crucial (5).

Recent studies suggest that dementia and fractures may be closely linked (6, 7). Although the primary function of the bone is traditionally thought to be as a structural scaffold to support the body and protect internal organs, emerging evidence indicates that the bone has a role in regulating whole-body metabolism as an endocrine organ (8–12). Bone-derived cells and modulators can be involved in the regulation of the nervous system, affecting brain health, which may be associated with the development of dementia (8–10). Furthermore, a range of effects of fractures on the body, such as systemic inflammation, chronic pain, and restricted movement, seem to theoretically influence the development of dementia (13, 14).

Identifying populations at high risk of dementia for early intervention is a critical strategy to address this global challenge. As the incidence of dementia is rising at an alarming rate and there is a large population base of fractures, any association between the two could have important implications for medical practice. Several observational studies have investigated the association between a history of fractures and dementia risk but yielded inconsistent evidence (15, 16). For example, the study by Hsu et al. showed hip fracture to be a predictor of dementia risk (15), while Barnes et al. found that the association between skull fracture and dementia risk did not reach statistical significance (16). Considering the lack of a study with a comprehensive overview of this topic, we, therefore, performed a meta-analysis and systematic review of published longitudinal observational studies to examine whether people with a history of fractures have an increased risk of dementia compared with the general population.

## Materials and methods

The present study was reported following the guidelines of the Preferred Reporting Items for Systematic Reviews and Meta-Analyses (PRISMA 2020) (17). The protocol for this study is not registered.

### Search strategy

We systematically searched the databases of PubMed, Web of Science, Embase, and the Cochrane Library for literature exploring the association between a history of fractures and the risk of dementia. The recent search date was 10 January 2023, and no filters were used. The search strategy included terms related to fracture and dementia, such as “fracture,” “dementia,” “Alzheimer disease.” The Medical Subject Headings terms and free-text phrases provided in each database were combined through Boolean operators. The specific search strategy for each database is provided in Supplementary Table 1.

### Study selection and eligibility criteria

The records generated from the database search were imported into EndNote X9 for review. After removing duplicates, the titles and abstracts of all the records were first screened to exclude reports that were clearly irrelevant to the study topic. Potentially eligible reports were then read in full to identify them for final inclusion. This process was done independently by two investigators (LS and XX), with disagreements resolved by consensus among all authors.

Inclusion criteria were as follows: (a) the study design was a longitudinal observational study exploring the association between any type of fracture and subsequent risk of dementia and (b) the study outcomes provided relative risk estimates for the sample with a history of fractures compared to controls, such as hazard ratio (HR), odd ratios (OR), risk ratio (RR), or provided sufficient data to calculate effect sizes. Exclusion criteria were as follows: (a) single-arm studies without a control group; (b) cross-sectional studies with a non-longitudinal design; (c) case reports; (d) study outcomes other than risk of dementia; (e) pre-clinical studies; and (f) articles that did not generate primary data, such as reviews, commentaries, and opinions.

### Data extraction

Information from eligible studies was extracted independently by two authors and cross-checked. The following data were extracted: first author, year of publication, study region, sample source, study design, sample size, mean age, method of identification of fracture and dementia, confounding factors considered, duration of follow-up, and effect estimates with corresponding 95% confidence intervals (CIs) for the risk of dementia in people with a history of fractures.

### Quality assessment

The quality of the included studies was assessed using the Newcastle-Ottawa Scale (NOS) in three main areas: participant selection, comparability between study groups, and outcome assessment in cohort study/exposure ascertainment in case-control studies (18). A NOS score of 7–9 was considered to be a high-quality study; otherwise, it was considered to be at a high risk of bias.

### Statistical analysis

All data analyses in this study were conducted using Stata MP/16.0. Effect estimates were pooled using fixed effects (Mantel-Haenszel) or random effects (DerSimonian-Laird) models according to the level of heterogeneity (19). We performed separate meta-analyses based on statistical measures, including HR, OR, and standardized incidence ratio (SIR). The level of heterogeneity was measured using Higgin's *I*^2^ statistic and Cochran's *Q* test (20). If both *I*^2^ <50% and *P* > 0.10 were met, the fixed-effects model was used; otherwise, there was significant heterogeneity, so the random-effects model was used to provide a more conservative estimate. Subgroup analyses were conducted based on the main characteristics of participants, including age, gender, fracture site, type of dementia, region, duration of follow-up, and study design. Sensitivity analyses were conducted by sequentially excluding a study and then re-running the pooled analysis. We also compared the results of the random-effects and fixed-effects models to examine the stability of the meta-analysis. The risk of publication bias was examined quantitatively by Egger's and Begg's tests. All tests were two-tailed, and the threshold for statistical significance was set at *P* < 0.05.

## Results

### Study selection

**Figure 1:**
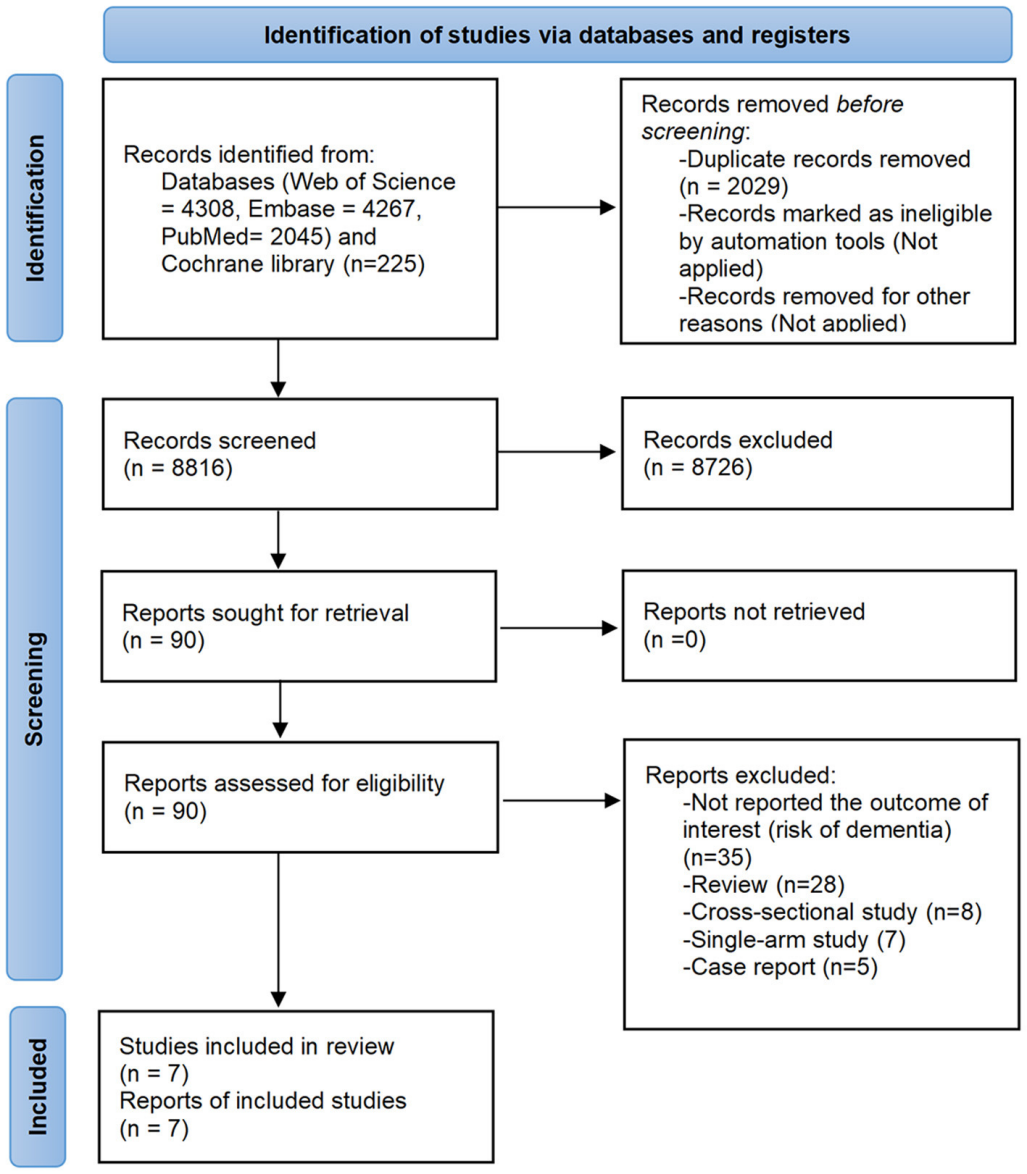
Flow diagram of the study selection process.

The database search yielded a total of 10,845 records. After screening according to pre-defined eligibility criteria, seven studies were eventually identified for qualitative and/or quantitative analysis (15, 16, 21–25). The screening process is shown in Figure 1.

### Study characteristics

The seven eligible studies comprised 3,658,108 participants, of whom 136,179 were patients with a history of fractures (15, 16, 21–25). The study samples were from China (*n* = 3), Korea (*n* = 1), the USA (*n* = 1), the UK (*n* = 1), and Denmark (*n* = 1). The mean age of the participants was over 50 years, and the follow-up period ranged from 9 to 15 years. Six studies were cohort studies, five of which used HR as the statistical measure (15, 16, 21, 22, 24) and one of which used SIR (23); the remaining one was a case-control study with OR as the measure (25). One study was a prospective design, and the other six were retrospective studies. Dementia and fracture cases were identified primarily by the International Classification of Diseases coding system. Details of each study are presented in Table 1.

**Table 1:** Characteristics of included studies.

**References**	**Year**	**Region**	**Sample source**	**Study design**	**Fracture cases**	**Controls**	**Mean age, year**	**Identification of Fracture**	**Identification of Dementia**	**Confounding factors adjusted**	**Follow-up, year**
Shang et al. (21)	2022	UK	UK Biobank	Retrospective cohort study	2,104	469,381	56.8	ICD codes; self-reported fields;	ICD codes; self-reported fields;	Age, genders, education, income, BMI, smoking, physical activity, alcohol consumption, sleep duration, diet, blood pressure, HDL-C, LDL-C, triglycerides, and HbA1c.	Median 11.9
Fann et al. (22)	2018	Denmark	Danish Civil Registration System	Prospective cohort study	7,006	2,662,759	80.7	ICD codes	ICD codes; ATC codes	Age, genders, marital status, calendar period, medical and neurological comorbidities (diabetes, ischemic heart disease, congestive heart failure, atrial fibrillation or flutter, peripheral vascular disease, cerebrovascular disease, autoimmune disease, HIV, Parkinson's disease, and epilepsy), psychiatric comorbidities (depression, bipolar disorder, schizophrenia, and substance abuse).	Mean 9.9
Yang et al. (23)	2019	China	NHIRD	Retrospective cohort study	23,890	Standardized incidence ratio	68.7	ICD codes	ICD codes	Age, genders	1–10
Tsai et al. (24)	2014	China	NHIRD	Retrospective cohort study	66,797	133,594	51.1	ICD codes	ICD codes	Age, genders, urbanization, and co-morbidities of diabetes, hypertension, stroke, CAD, head injury, depression and cognitive impairment	1–12
Kim et al. (25)	2020	Korea	NHIS-NSC	Retrospective case-control study	9,699	42,236	>60	ICD codes	ICD codes	Age, genders, income, region of residence, and history of hypertension, diabetes, dyslipidemia, ischemic heart disease, cerebral stroke, depression, osteoporosis, distal radius fracture, hip fracture, and spine fracture.	1–11
Hsu et al. (15)	2022	China	Clinical Data Analysis and Reporting System	Retrospective cohort study	26,424	26,424	81.4	ICD codes	ICD codes	Age, genders, calendar year on index date, institution cluster, length of hospital stay, cardiovascular diseases, respiratory-related diseases, endocrine and metabolic disorders, renal diseases, liver diseases, bone-related diseases, depression, connective tissue disease, medication history	Median: fracture: 4.9; control 5.0
Barnes et al. (16)	2014	USA	VHA National Patient Care Database	Retrospective cohort study	259	187,535	>55	ICD codes	ICD codes	None	Mean 7.4

NHIRD, Taiwan National Health Insurance Research Database; NHIS-NSC, Korean National Health Insurance Service-National Sample Cohort; VHA, Veterans Health Administration; ICD, International Classification of Diseases Coding System; ATC, Anatomical Therapeutic Chemical Classification System.

### Risk of bias assessment

Except for one study by Barnes et al. with a NOS score of 6, the NOS scores of the included studies were between 8 and 9, indicating a high level of overall quality of evidence (Table 2). The sample of the study by Barnes et al. was derived from the Veterans Health Administration National Patient Care Database and did not report risk of dementia adjusted for confounders, so the representativeness of the sample and comparability between exposed and unexposed cohorts were considered inadequate (16). All studies had clear definitions for exposure and outcome and sufficient lengths of follow-up.

**Table 2:** The quality assessment of included studies according to the Newcastle-Ottawa Rating Scale.

**Cohort Study**	**Representativeness of exposed cohort**	**Selection of non-exposed cohort**	**Ascertainment of exposure**	**Outcome not present before study**	**Comparability**	**Assessment of outcome**	Follow-up long enough^*^	**Adequacy of follow-up**	**Quality score**
Shang et al. (21)	⋆	⋆	⋆	⋆	⋆⋆	⋆	⋆	⋆	9
Fann et al. (22)	⋆	⋆	⋆	⋆	⋆⋆	⋆	⋆	⋆	9
Yang et al. (23)	⋆	⋆	⋆	⋆	⋆✰	⋆	⋆	⋆	8
Tsai et al. (24)	⋆	⋆	⋆	⋆	⋆⋆	⋆	⋆	⋆	9
Hsu et al. (15)	⋆	⋆	⋆	⋆	⋆⋆	⋆	⋆	⋆	9
Barnes et al. (16)	✰	⋆	⋆	⋆	✰✰	⋆	⋆	⋆	6
**Case-control study**	**Case definition**	**Representativeness of the cases**	**Selection of Controls**	**Definition of Controls**	**Comparability**	**Ascertainment of exposure**	**Same method of ascertainment**	**Non-response rate**	**Quality score**
Kim et al. (25)	⋆	⋆	⋆	⋆	⋆⋆	⋆	⋆	⋆	9

^*^A star was assigned for a maximum follow-up period of more than 5 years.

### Overall association between history of fracture and risk of dementia

**Figure 2:**
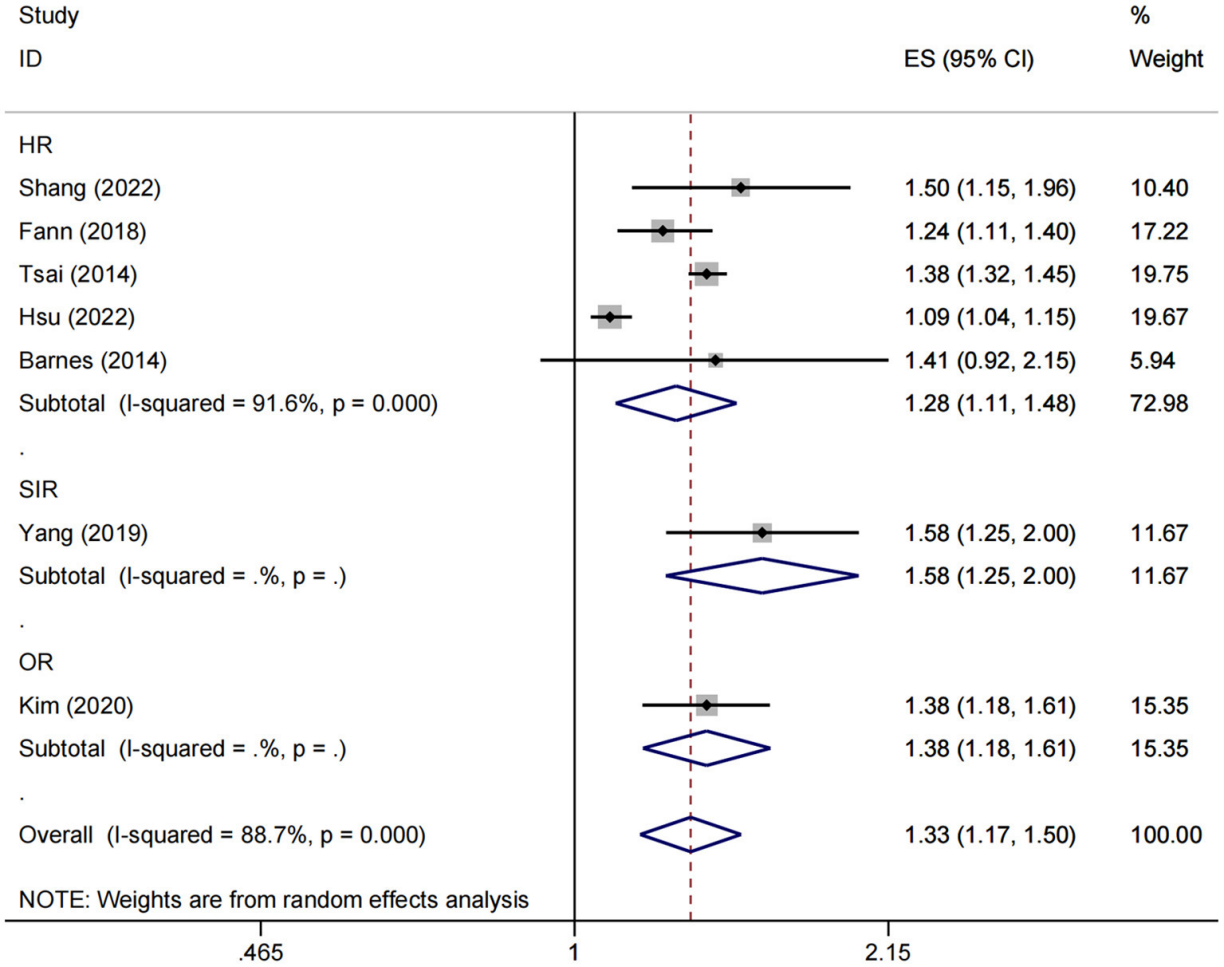
Overall association between fracture and subsequent risk of dementia. ES, effect size; HR, hazard ratio; OR, odds ratio; SIR, standardized incidence ratio.

Seven studies involving over 3 million participants evaluating the risk of dementia in patients with a history of fractures were included in the meta-analysis. To avoid confusion in the interpretation of the results, we performed separate analyses according to different statistical measures. For the cohort studies with HR endpoints, Higgin's *I*^2^ statistics and Cochran's *Q* tests indicated significant heterogeneity between studies (*I*^2^ = 91.6%, *P* < 0.001), and therefore the random-effects model was used. The pooled results showed a significant association between fracture and subsequent risk of dementia (HR = 1.28, 95% CI: 1.11–1.48) (Figure 2).

Moreover, both SIR and OR endpoints were reported in only one study (23, 25), with 1.58 (95% CI: 1.25–2.00) and 1.38 (95% CI: 1.18–1.61), respectively (Figure 2).

### Subgroup analysis

To examine differences in the association between history of fractures and dementia risk in samples with different characteristics, we conducted a stratified analysis according to fracture site, age, gender, region, follow-up time, study design, and dementia subtype (Table 3).

**Table 3:** Stratified analysis of the association between history of fractures and dementia risk in cohort studies.

**Subgroup**	**Studies (*n*)**	**HR (95% CI)**	***P*_overall effect_**	**Heterogeneity (*I^2^*, *P_*Q*_*)**	**Model**
**Fracture site**					
Skull fracture	2	1.25 (1.12–1.40)	<0.001	0.0%, 0.567	Fixed-effects
Vertebrae fracture	1	1.47 (1.12–1.93)	0.006	NA	NA
Upper limb fracture	1	1.29 (1.15–1.44)	<0.001	NA	NA
Hip fracture	2	1.32 (0.90–1.92)	0.152	97.3%, <0.001	Random-effects
Thigh/leg/ankle fracture	1	1.35 (1.18–1.55)	<0.001	NA	NA
Multiple fractures	1	1.34 (1.26–1.43)	<0.001	NA	NA
**Dementia subtype**					
Alzheimer's disease	3	1.27 (1.02–1.58)	0.03	57.0%, 0.10	Random-effects
Vascular dementia	1	1.83 (1.10–3.04)	0.02	NA	NA
**Gender**					
Female	2	1.25 (1.06–1.46)	0.006	92.2%, <0.001	Random-effects
Male	2	1.17 (0.78–1.78)	0.446	97.5%, <0.001	Random-effects
**Age**					
<65 years	1	1.84 (1.21–2.78)	0.004	NA	NA
≥65 years	2	1.23 (0.97–1.55)	0.084	97.4%, <0.001	Random-effects
**Region**					
Asia	2	1.23 (0.97–1.55)	0.083	97.8%, <0.001	Random-effects
Europe	3	1.29 (1.16–1.43)	<0.001	0.0%, 0.40	Fixed-effects
**Followed-up**					
<5 years	1	1.46 (1.37–1.56)	<0.001	NA	NA
≥5 years	2	1.32 (1.23–1.41)	<0.001	0.0%, 0.88	Fixed-effects
**Study design**					
Prospective study	1	1.24 (1.11–1.40)	<0.001	NA	NA
Retrospective study	4	1.30 (1.08–1.56)	0.005	93.7%, <0.001	Random-effects
**Study quality**					
NOS≥7	4	1.27 (1.09–1.48)	0.003	93.6%, <0.001	Random-effects
NOS <7	1	1.41 (0.92–2.15)	0.113	NA	NA

HR, hazard ratio; NA, not applicable.

For the HR endpoint, results showed that almost all sites of fracture were significantly associated with higher subsequent dementia risk, including skull (HR = 1.25, 95% CI: 1.12–1.40), vertebrae (HR = 1.47, 95% CI: 1.12–1.93), upper limb (HR = 1.29, 95% CI: 1.15–1.44), and thigh/leg/ankle (HR = 1.35, 95% CI: 1.18–1.55) fractures. For hip fractures, the direction was consistent, although not significant (HR = 1.32, 95% CI: 0.90–1.92). The risk was elevated for both Alzheimer's disease (HR = 1.27, 95% CI: 1.02–1.58) and vascular dementia (HR = 1.83, 95% CI: 1.10–3.04). Stratified analysis based on follow-up time showed that after excluding cases of dementia that occurred during the first 5-year follow-up period, patients with a history of fractures also had a significantly higher risk of long-term dementia beyond 5 years (HR = 1.32, 95% CI: 1.23–1.41). Tsai et al. found that even 9 years after the fracture, the risk of dementia remained higher compared to the non-fracture sample (HR = 1.30, 95% CI: 1.11–1.53) (24). No gender, age, or region specificity was observed; although statistical significance disappeared in some subgroups, the direction remained consistent. No significant changes were observed in the pooled results after exclusion of low-quality studies (HR = 1.27, 95% CI: 1.09–1.48). Findings from prospective and retrospective studies were similar (Table 3).

Two studies reported the SIR and OR of dementia risk in patients with a history of fractures, respectively (23, 25). A case-control study from the Korean national health insurance database showed that spine (OR = 1.31, 95% CI: 1.22–1.41), distal radius (OR = 1.23, 95% CI: 1.10–1.37), and hip (OR = 1.64, 95% CI: 1.48–1.83) fractures were all significantly associated with a subsequent increased risk of dementia; no age or sex specificity was observed (25). Another retrospective cohort study from the Taiwan region showed that individuals with facial bone fractures also had a higher risk of subsequent dementia (SIR = 1.58, 95% CI: 1.25–2.00); no further stratification analysis was performed (23).

### Sensitivity analysis

**Figure 3:**
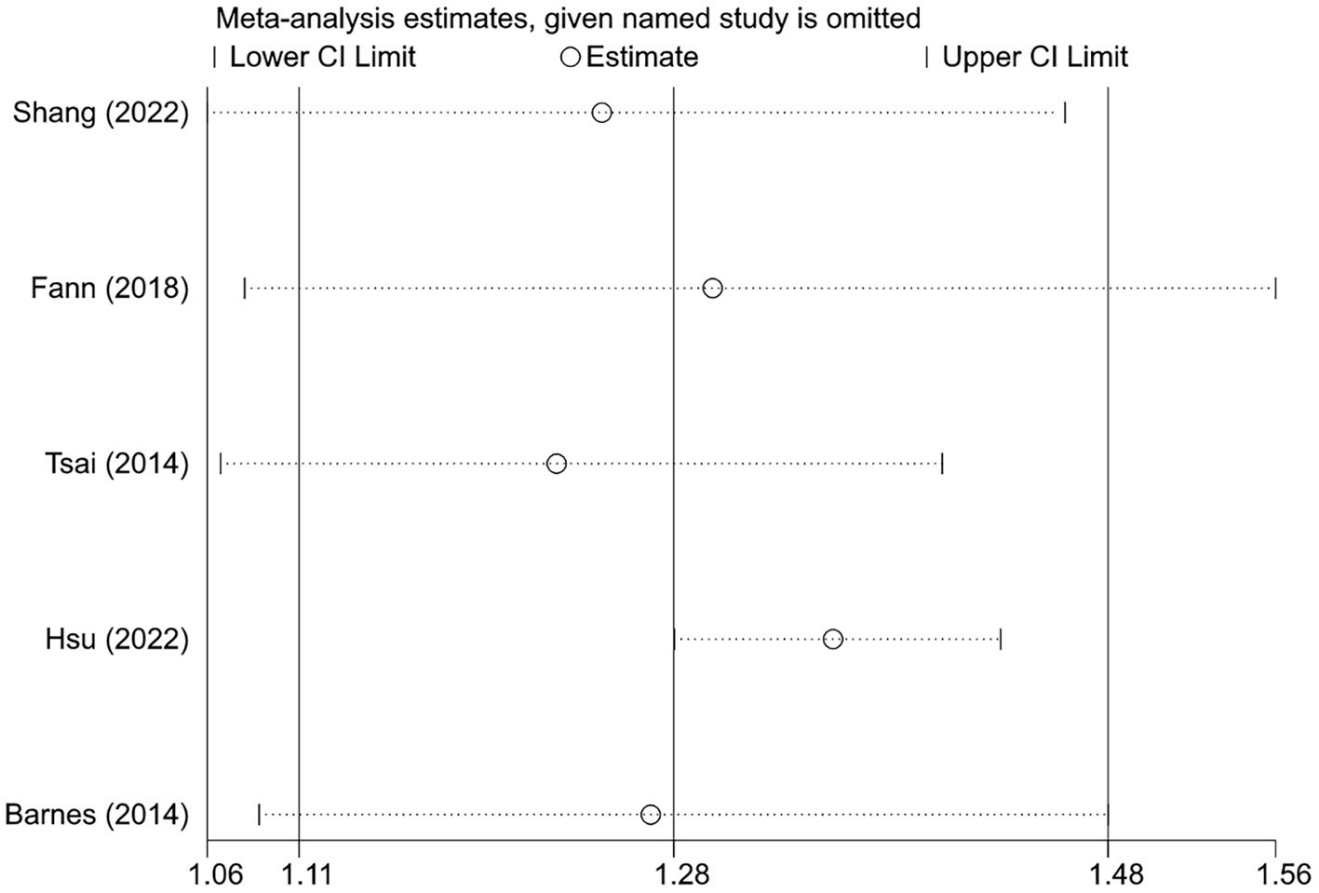
The effects of the individual studies on the overall pooled result in cohort studies.

Since there was only one study for the SIR and OR endpoints and no pooled analysis was performed, sensitivity analysis was not applicable. For the HR endpoint, sensitivity analysis was conducted by comparing the results of the random-effects and fixed-effects models. As shown in Table 4, the results of the two models were comparable, indicating that the current findings were relatively robust. In addition, we also examined whether the pooled results were dominated by individual studies by sequentially excluding one study and then re-estimating the effect size. As shown in Figure 3, the direction and significance of effect estimates remained unchanged following the exclusion of any individual study; the results of the subgroup analyses were also consistent in the leave-one-out analysis, although significance disappeared in several subgroups (data not shown).

**Table 4:** Sensitivity analysis by comparison of random-effects and fixed-effects model results in cohort studies.

**Analysis groups**	**HR (95% CI), random-effects model**	**HR (95% CI), fixed-effects model**
Total	1.28 (1.11–1.48)	1.24 (1.20–1.28)
Skull fracture	1.25 (1.12–1.40)	1.25 (1.12–1.40)
Vertebrae fracture	1.32 (1.23–1.42)	1.32 (1.23–1.42)
Upper limb fracture	1.29 (1.15–1.44)	1.29 (1.15–1.44)
Hip fracture	1.32 (0.90–1.92)	1.16 (1.11–1.22)
Thigh/leg/ankle fracture	1.35 (1.18–1.55)	1.35 (1.18–1.55)
Multiple fractures	1.34 (1.26–1.43)	1.34 (1.26–1.43)
Alzheimer's disease	1.27 (1.02–1.58)	1.22 (1.07–1.39)
Vascular dementia	1.83 (1.10–3.04)	1.83 (1.10–3.04)
Female	1.25 (1.06–1.46)	1.24 (1.19–1.30)
Male	1.17 (0.78–1.78)	1.22 (1.14–1.30)
<65 years	1.84 (1.21–2.78)	1.52 (1.34–1.72)
≥65 years	1.23 (0.97–1.55)	1.22 (1.17–1.26)
Followed-up <5 years	1.46 (1.37–1.56)	1.46 (1.37–1.56)
Followed-up ≥ 5 years	1.32 (1.23–1.41)	1.32 (1.23–1.41)
Asia	1.23 (0.97–1.55)	1.24 (1.19–1.28)
Europe	1.29 (1.16–1.43)	1.29 (1.16–1.43)
Prospective study	1.24 (1.11–1.40)	1.24 (1.11–1.40)
Retrospective study	1.30 (1.08–1.56)	1.24(1.20–1.28)
NOS ≥ 7	1.27 (1.09–1.48)	1.24 (1.20–1.28)
NOS <7	1.41 (0.92–2.15)	1.41 (0.92–2.15)

### Evaluation of publication bias

No risk of publication bias was detected by Egger's and Begg's tests with *P*-values of 0.812 and 1.000, respectively.

## Discussion

Through quantitative and qualitative analysis of the current evidence, we found a 28% increased risk of dementia in patients with a history of fractures, with a similar increase observed at almost all sites of fracture. No gender, age, region, duration of follow-up, study quality, and study design specificity were observed. The results from case-control studies were similar.

Current research suggests that people who experience fractures are at a higher risk of developing dementia in the future than the general population. Whether as a predictor or a risk factor, this highlights the need to focus on the preventive management of dementia after a fracture. Multidisciplinary, including social, psychological, and medical, measures such as post-operative rehabilitation, mental health support, regular exercise, and nutritional supplementation, may be worth considering (26). In addition, further exploration of the factors influencing the risk of dementia in the population with a history of fractures to develop more precise interventions and optimize care is urgent.

There are no mechanistic studies that suggest that fractures directly cause dementia. Some of the physical changes that occur after a fracture, such as inflammatory responses, complications from fracture surgery, and reduced quality of life after a fracture, may contribute to cognitive decline and dementia risk. There is a prolonged inflammatory response during fracture healing, and a range of inflammatory cytokines, including interleukin (IL)-2, IL-6, IL-8, tumor necrosis factor-alpha, and C-reactive protein, are elevated (27, 28); previous studies have shown that these pro-inflammatory cytokines are associated with an increased risk of dementia (29, 30). In addition, oxidative stress during fracture healing produces excess superoxide radicals, which may increase the risk of vascular dementia and Alzheimer's disease (31, 32). Prospective studies have found that inflammatory cell products are elevated after fracture not only in peripheral blood but also in cerebrospinal fluid (33, 34). Anesthesia for surgery, such as general anesthesia, has been found to have a possible link with postoperative cognitive impairment (35). Delirium after fracture surgery has also been associated with a significantly higher risk of dementia (36). Changes in quality of life after fracture, such as chronic pain, impairment of social engagement due to changes in appearance, impaired balance function, and reduced physical activity, may all impair cognition and contribute to dementia (37). Fractures, particularly of the lower limbs, are an independent influencing factor in the short- and long-term functional decline of older people, irrespective of health conditions before the fracture (38).

To the best of our knowledge, this is the first study to systematically analyze the risk of dementia in people with a history of fractures using a meta-analysis approach in both cohort and case-control studies separately. Although a causal relationship between fracture and dementia risk cannot be established based on the current study, this meta-analysis provides relatively robust evidence for an increased risk of dementia in people with a history of fractures. The level of quality of the included studies was generally high, and a meta-analysis of over three million participants can be considered a rigorous estimate.

The inherent limitations of the included observational studies limit the ability to conclude that there is a causal effect of fracture on dementia. Reverse causality is one of the major biases introduced by observational studies. As a chronically progressive neurodegenerative disease, the prodromal phase of dementia may last several years (1). Previous studies have shown that patients with dementia are at significantly higher risk of fracture, suggesting that the current topic is susceptible to confounding by reverse causation (39). However, published studies have taken the approach of excluding participants who developed dementia at early follow-up, which effectively reduces the impact of pre-existing but undiagnosed cases of dementia before the fracture on risk estimates (15, 21, 24). Stratified analysis showed that the risk of dementia remained significantly higher more than 5 years after fracture (HR = 1.32, 95% CI: 1.23–1.41, *P* < 0.001); Tsai et al. found that patients with fractures still had a higher risk of long-term dementia 9 years later than non-fractured individuals (24). In addition, Hsu et al. conducted a sensitivity analysis excluding participants who were nursing home residents, as preexisting but undiagnosed dementia was thought to be more prevalent in patients admitted through nursing homes; their results were similar to the current meta-analysis (15). These findings suggest that the current results are less likely to be subject to reverse causal confounding. If the hypothesis of a significantly higher risk of dementia in people with a history of fractures holds, it implies a bidirectional association between fractures and dementia.

Unadjusted confounders are another important bias that affects the reliability of observational studies. Both dementia and fractures occur most commonly in older patients, which may be subject to confounding by shared exposures. Frailty-related factors are evident as potential confounders; for example, obesity, micronutrient deficiency, low educational level, lack of social support, chronic disease, and lifestyle factors are all strongly associated with the development of both fractures and dementia (40, 41). Despite many efforts to correct for potential confounders in published studies, there is still bias due to residual and unconsidered confounders. Interestingly, the study by Hsu et al. established fracture and control cohorts in people admitted to hospitals because of an accidental fall (15). This may help to reduce the effect of frailty-related confounders to some extent compared to studies using the general population as the sample source, as falls and frailty are closely correlated. Their results still showed a significant association between fracture and subsequent risk of dementia, but the effect size was relatively low (HR = 1.09, 95% CI 1.04–1.15, *P* < 0.001), suggesting that studies using the general population as controls may have overestimated the results due to frailty-related factors (15).

There are several limitations to the current meta-analysis that need to be noted. First, almost all studies identified cases by disease diagnosis codes, which inevitably leads to underdiagnosis, misdiagnosis, and delayed diagnosis. Second, data from most of the current studies were collected retrospectively and from health insurance administrative claims databases, which are less complete than prospective studies and have residual confounding factors, particularly lifestyle factors such as smoking, alcohol consumption, and physical activity. It was therefore impossible to establish a causal relationship. Third, the majority of participants were older than 50 years, and therefore the association between a history of fractures and dementia risk in the young population is unclear. Fourth, although we conducted a stratified analysis, the level of heterogeneity in most results of the meta-analysis remained high, which may reflect differences in medical practice and environmental factors across regions. Therefore, we used the random-effects model for effect estimation to provide more conservative results, which accounts for the disappearance of statistical significance in some subgroups. Fifth, in some studies exploring the association between specific fractures and dementia, the control group did not exclude patients with other fracture types. If the finding of a positive association between history of fractures and dementia risk suggested by the current meta-analysis holds, then these studies would underestimate the effect size.

## Conclusion

This meta-analysis based on population studies suggests that people with a history of fractures are at increased risk of developing dementia in the future. Fractures may be one of the risk factors or predictors of dementia, and combining genetics, lifestyle, and other environmental risk factors may improve dementia prevention and screening work.

## Data availability statement

The original contributions presented in the study are included in the article/Supplementary material, further inquiries can be directed to the corresponding author.

## Author contributions

YLi and LS: conception and design. LS, YLia, and XX: acquisition of data. LS and XL: statistical analysis. LS, YLia, and XX: drafting of the manuscript. YLi: critical revision of the manuscript. All authors contributed to the article and approved the submitted version.
